# Phospholipase A_2_ from *Daboia siamensis* venom induces acute kidney injury: involvement of ion channels in an isolated perfused rabbit kidney model

**DOI:** 10.1590/1678-9199-JVATITD-2025-0016

**Published:** 2025-08-04

**Authors:** Narongsak Chaiyabutr, Taksa Vasaruchapong, Panithi Laoungbua, Orawan Khow, Lawan Chanhome, Visith Sitprija

**Affiliations:** 1 Queen Saovabha Memorial Institute, Thai Red Cross Society, Pathumwan, Bangkok, Thailand.; 2 Snake Farm, Queen Saovabha Memorial Institute, Thai Red Cross Society, Bangkok, Thailand.; 3 Department of Research and Development, Queen Saovabha Memorial Institute, Thai Red Cross Society, Bangkok, Thailand.

**Keywords:** *Daboia siamensis* venom, Phospholipase A_2_, Acute kidney injury, Isolated perfused rabbit kidney, Ion channels

## Abstract

**Background:**

Acute kidney injury (AKI) is a serious complication associated with *Daboia siamensis* envenomation, primarily due to direct nephrotoxicity. This study aimed to investigate the effects of the phospholipase A_2_ (RvPLA₂) fraction from *D. siamensis* venom on renal function and to assess whether pretreatment with ion channel blockers could mitigate these effects using an isolated perfused kidney (IPK) model.

**Methods:**

Twenty IPKs were allocated into five groups (n = 4 each): (1) RvPLA₂ in calcium-deficient modified Krebs-Henseleit solution (MKHS), (2) RvPLA₂ in standard MKHS, (3) RvPLA₂ following pretreatment with verapamil (a voltage-gated Ca²⁺ channel blocker), (4) RvPLA₂ following pretreatment with amiloride (a Na⁺ channel blocker), and (5) RvPLA₂ following pretreatment with minoxidil (a KATP channel opener). Renal function parameters were assessed accordingly.

**Results:**

Administration of 280 μg of RvPLA₂ in calcium-deficient MKHS caused no significant changes in renal function. In contrast, RvPLA₂ in standard MKHS (1.9 mM Ca²⁺) significantly increased perfusion pressure (PP), renal vascular resistance (RVR), and free water excretion (*p* < 0.05), while non-significant increases were observed in glomerular filtration rate (GFR), urinary flow rate (UF), osmolar clearance (C_osm_), and the fractional excretion of sodium (FE_Na⁺_) and potassium (FE_K⁺_). Verapamil alone caused significant increases in GFR and C_osm_ (*p* < 0.05) and non-significant increases in PP, RVR, UF, FE_Na⁺_, and free water excretion. Amiloride and minoxidil alone did not alter renal function. Pretreatment with verapamil, amiloride, or minoxidil failed to prevent the renal functional changes induced by RvPLA₂.

**Conclusions:**

The RvPLA_2_ activity requires Ca^2+^ for activation which may target distinct sites on the cell membrane, including ion channel receptors in nephrons. The effects of RvPLA_2_ on glomerular and renal tubular function are independent and cannot be modified by pretreatment with different ion channel blockers.

## Background

Acute kidney injury (AKI) is the most serious complication during envenomation by Russell’s viper (*Daboia siamensis*) [1]. The most common causes of AKI in snakebite envenomation are related to several factors, including hemodynamic disturbances, immunologic reactions, and direct nephrotoxicity [2]. Various mechanisms have been implicated in the pathogenesis of *D. siamensis* venom-induced AKI, such as renal vascular obstruction by fibrin microthrombi (disseminated intravascular coagulation - DIC), ischemia or hypoperfusion caused by a drop in blood pressure, and pigment nephropathy resulting from hemolysis [3]. However, direct nephrotoxicity remains insufficiently demonstrated [4]. The role of proteolytic enzymes and vasoactive substances, which may promote or potentiate coagulation processes in renal tissues, cannot be excluded.

The etiology of *D. siamensis* venom-induced acute renal failure in humans and experimental animals is still not fully understood but likely involves a direct nephrotoxic effect of venom components on renal vascular and epithelial cells. The underlying renal mechanisms remain poorly characterized [4]. Impaired glomerular filtration and renal blood flow were observed in an *in vivo* study in which *D. siamensis* venom was injected intravenously into experimental dogs [5]. Additionally, *ex vivo* studies have shown that administering *D. siamensis* venom to isolated rabbit kidneys induces direct cytotoxic effects on renal tubular epithelial cells, reduces perfusion pressure, and increases vascular resistance [6, 7]. The varying responses observed in envenomation studies are likely due to significant differences in the toxin profiles of Russell’s viper venoms, which affect the clinical outcomes in snakebite victims [8].

The venom of *D. siamensis* contains a wide array of proteins, including both enzymatic and non-enzymatic components [9]. Many of these proteins are toxic and harmful to victims. However, the biological and pathological roles of specific venom components, such as phospholipase A₂ - a major component of *D. siamensis* venom (RvPLA₂) - in renal function remain to be clarified. RvPLA₂ toxins have been shown to initiate kidney injury by increasing renal vascular resistance and causing renal ischemia, thereby decreasing renal blood flow, glomerular filtration rate, and urine output. This toxin also promotes the release of inflammatory mediators in experimental dogs [10].

Several studies have characterized PLA₂ as a catalytic enzyme that induces acute local inflammatory reactions [11] through the hydrolysis of fatty acids at the sn-2 position of phospholipid membranes. This process involves phospholipid metabolism, signal transduction, and other essential cellular functions [12]. Phospholipases A₂ (PLA₂) are enzymes that catalyze the hydrolysis of cell membrane phospholipids at different positions, releasing fatty acids and lysophospholipids [13, 14]. Although most PLA₂ enzymes characterized to date require Ca²⁺ for activation, the biological and pathological effects of RvPLA₂ from viper venoms on kidney dysfunction - particularly with respect to ion channels in renal cells - have not yet been elucidated [15].

Understanding the mechanisms underlying AKI, including the association between ion channels and PLA₂ activity in the kidney, is crucial for determining the localization and extent of kidney injury during envenomation. It is well known that kidney function in different regions regulates blood ionic composition through the filtration and reabsorption of essential ions, secretion of excess ions, and conservation of water to concentrate urine. Some of these processes involve specific ion channels that play critical roles.

Most ion channels are pore-forming proteins found in the membranes of renal cells, particularly in distinct regions of the glomerulus and renal tubules within the nephron, as well as in intracellular organelles such as mitochondria, the endoplasmic reticulum, and the nucleus. Alterations in these channels or related transporters may lead to cellular and organ dysfunction. In the kidney, malfunctioning ion channels impair ion reabsorption, disrupt osmotic balance, and ultimately affect the body’s overall ionic homeostasis. However, clear evidence regarding the mechanisms by which RvPLA₂ affects ion channels in both vascular and tubular cell membranes - and its impact on renal function - is still lacking.

To investigate this, we conducted an *ex vivo* study using an isolated perfused rabbit kidney (IPK), a valuable model for characterizing the direct effects of RvPLA₂ on both vascular and tubular components of kidney function. This model eliminates higher-order influences, such as physical factors (including hydrostatic and osmotic pressures), the sympathetic nervous system, blood pressure, coagulation, and other blood-borne factors such as corpuscles and hormones, which may contribute to synergistic or secondary effects following PLA₂ activation [13]. A key advantage of the IPK model is its ability to isolate the direct renal effects of RvPLA₂ without interference from systemic feedback mechanisms.

In this study, we aimed to elucidate the effects of RvPLA₂, which is responsible for renal dysfunction, specifically in relation to changes in ion channel receptor activity in renal cells. These effects were investigated using the IPK model to determine whether pretreatment with ion channel blockers could ameliorate kidney injury caused by RvPLA₂. To assess this, we recorded the effects of RvPLA₂ in kidneys pretreated with verapamil (a voltage-gated Ca²⁺ channel blocker), amiloride (a Na⁺ channel blocker), or minoxidil (a KATP channel opener). Furthermore, RvPLA₂ specifically catalyzes the hydrolysis of cell membranes and may influence ion channel function in both renal vascular and epithelial cells during acute kidney injury. As a result, the findings from ion channel blocker pretreatments followed by sequential addition of RvPLA₂ in the same IPK model may be of broader scientific interest.

## Methods

## Animals 

Adult male white New Zealand rabbits, weighing 2-3 kg, were obtained from the Animal House of the Queen Saovabha Memorial Institute. The animals were housed in stainless steel cages and received a standard diet and water. They were exposed to a 12 h light/dark cycle and maintained at a laboratory temperature of 26 ± 1 °C. All animals were quarantined for 14 days before the experiments. The study was conducted in accordance with ethical guidelines and approved by the Ethics Committee of the Queen Saovabha Memorial Institute (Animal Care and Use approval number QSMI-ACUC-03-2016), following the guidelines of the National Research Council of Thailand.

### Venom and chemical analyses

*Venom*

Lyophilized *Daboia siamensis* venom was provided by the Queen Saovabha Memorial Institute, Thai Red Cross Society. A pooled venom sample was obtained from 14 adult male and female Russell’s vipers (*D. siamensis*) originating from the eastern region of Thailand and maintained at the Snake Farm, Queen Saovabha Memorial Institute. The crude venom was stored at -20 °C under permission from the National Control Laboratory for Biological Products, Thailand (Permit No. 0392-40/57). The venom was subsequently fractionated to isolate RvPLA₂.

*Ion channel blockers*

The following ion channel blockers were used: verapamil hydrochloride (Isoptin; Abbott Laboratories, Ireland), amiloride hydrochloride, and minoxidil (both from Sigma-Aldrich, Saint Louis, MO, USA).

*Isolation of phospholipase A*_*2*_ *from the lyophilized* Daboia siamensis *venom (RvPLA*_*2*_ *)*

The PLA₂ was isolated and characterized from *D. siamensis* venom. Lyophilized venom (20 mg/mL, w/v) was dissolved in buffer A (50 mM phosphate buffer, pH 6.0). After centrifugation at 10,000 rpm for 5 min, the supernatant was applied to ion-exchange chromatography using a HiTrap CMFF column (GE Healthcare, Sweden). The column was washed with 5 volumes of buffer A, and elution was carried out with a linear NaCl gradient (0-1 M) in buffer A at 0.5 mL/min. Fractions (1 mL each) were collected and monitored at 280 nm using Unicorn 6.3 Software on an ÄKTA Pure FPLC system (GE Healthcare, Sweden). Four peaks were detected, and those with PLA₂ activity were pooled and further purified via size-exclusion chromatography using a Superdex™ 75 10/300 GL column (GE Healthcare, Sweden) equilibrated with 10 mM PBS (pH 7.4). Elution was performed at room temperature with a flow rate of 0.5 mL/min, and 1 mL fractions were collected. Protein concentration was determined at 280 nm, and the active fractions were named RvPLA₂. For the study, 1 mL of RvPLA₂ at 280 μg/mL was prepared in PBS (0.12 M NaCl, 0.04 M sodium phosphate buffer, pH 7.2). This dose was selected and adjusted according to Chaiyabutr et al. [7].

### Experimental design

*Isolated perfused kidney preparation*

The preparation of the isolated perfused kidney (IPK) followed previously described methods [6]. Briefly, adult male rabbits were fasted for 24 h with free access to water. Anesthesia was induced using pentobarbital sodium (30 mg/kg, i.v., via the marginal ear vein), and 1,000 units of heparin were administered intravenously. After laparotomy, the left ureter was cannulated with a polyvinyl catheter. The left renal artery was carefully dissected and ligated. A 19-gauge stainless-steel needle (1.0 inch, smooth tip) was used for arterial cannulation. The artery was flushed with heparinized saline (100 units/mL), and the kidney was excised with the renal vein and ureter intact.

The isolated kidney was transferred to a thermostatically controlled tissue bath organ chamber (Radnoti, catalogue No. 166070, Grass Technologies, Monrovia, CA, USA). A recirculating *ex vivo* perfusion system was employed, using oxygenated modified Krebs-Henseleit solution (MKHS) at 37 °C, aerated with a gas mixture (O₂:CO₂, 95:5), and pumped via a rotary pump (EYELA RP-1000). The perfusion flow rate was maintained at 40-60 mL/min.

The MKHS composition per 100 mL was: 141 mM Na⁺, 5.4 mM K⁺, 1.9 mM Ca²⁺, 2.4 mM Mg²⁺, 126 mM Cl⁻, 25 mM HCO₃⁻, 2.44 mM SO₄²⁻, 1.5 mM PO₄³⁻, and 13 mM amino acids (individual concentrations listed as in original text) [16]. The perfusate also contained 100 mg D-glucose, 50 mg inulin, 3 g bovine serum albumin (BSA fraction V; Sigma), and 2 g dextran (Sigma) as oncotic agents. The pH was adjusted to 7.4. Perfusion pressure was measured via the cannula tip using a manometer or pressure transducer and recorded with a physiograph (Polygraph Model 79, Grass Instruments Co.). After initiating perfusion, the kidney was allowed to equilibrate for 30 min, during which urine flow and perfusion pressure were stabilized at 100 mmHg. Measurements of perfusate and urine were collected every 3 min.

**Ex vivo *studies of RvPLA***_*2*_ ***and ion channel blockers***

This experiment aimed to investigate the effects of RvPLA₂ on renal function and whether these effects could be modified by pretreatment with specific ion channel blockers in the rabbit IPK model. After a 30 min equilibration period, perfusion was divided into three phases: 15 min internal control, 15 min ion channel blocker pretreatment, and 30 min RvPLA₂ treatment. Measurements were taken every 3 min for perfusion pressure, perfusate, and urine analysis.

Preparations were divided into five groups (n = 4 per group):

- Group 1 (Ca²⁺-deficient MKHS + RvPLA₂): MKHS without CaCl₂. RvPLA₂ (280 μg/mL, 1 mL) was added after 30 min. Renal functions were measured before and 30 min after RvPLA₂ treatment.
- Group 2 (MKHS + RvPLA₂): standard MKHS. RvPLA₂ (280 μg/mL, 1 mL) was added after 30 min. Renal functions were measured before and after RvPLA₂ treatment.
- Group 3 (verapamil + RvPLA₂): verapamil (4 mg/100 mL) was added at 15 min. Effects were assessed for 15 min, followed by RvPLA₂ addition (280 μg/mL) at 30 min. Renal functions were evaluated after each phase.
- Group 4 (amiloride + RvPLA₂): amiloride (500 μg/100 mL) was added at 15 min, followed by RvPLA₂ (280 μg/mL) at 30 min. Renal functions were measured accordingly.
- Group 5 (minoxidil + RvPLA₂): minoxidil (5 mg/100 mL) was added at 15 min, followed by RvPLA₂ (280 μg/mL) at 30 min. Renal functions were recorded after each phase.

### Measurements and calculation of renal functions 

Renal function was assessed using perfusate (P) and urine (V) samples. Inulin concentration was measured using a modified anthrone method [17]. Na⁺ and K⁺ concentrations were determined with a flame photometer (BWB Technologies, UK), and osmolality was measured with an osmometer (Fiske® Micro-osmometer Model 210, Fiske^®^ Associates, USA).

Renal clearance (C) was calculated as C = UV/P (U: urine concentration, V: urine flow rate, P: perfusate concentration). Inulin clearance (C_in_) was used to estimate GFR. Osmolar clearance (C_osm_) was calculated as U_osm_ × V/P_osm_. Fractional excretion of sodium (FE_Na⁺_) and potassium (FE_K⁺_) were calculated as C_Na⁺_/C_in_ and C_K⁺_/C_in_, respectively. Free water excretion was the ratio V/GFR. Renal vascular resistance (RVR) was calculated from perfusion pressure (PP) and flow rate: RVR = PP/flow rate.

Percentage changes (%DV) were calculated as: %DV = [(CV - CB)/CV] × 100, or %DV = [(CV - CB×RvPLA₂)/CV] × 100, where DV is value change, CV is control value, CB is ion channel blocker value, and CB × RvPLA₂ is the value after RvPLA₂ addition.

### Statistical analysis 

Data are presented as mean ± SEM for n = 4 IPKs per group. Statistical analyses were performed using Prism 5.0 (GraphPad Software, San Diego, CA, USA). One-way repeated measures ANOVA with Bonferroni post hoc test was used to compare values across time points. Paired *t*-tests were used to compare maximal percentage responses between periods (control vs. pretreatment or treatment). A *p*-value < 0.05 was considered statistically significant.

## Results 

### Effect of RvPLA_2_ on the renal function in rabbit IPK with Ca^2+^-deficient MKHS perfusion

**Figure 1:**
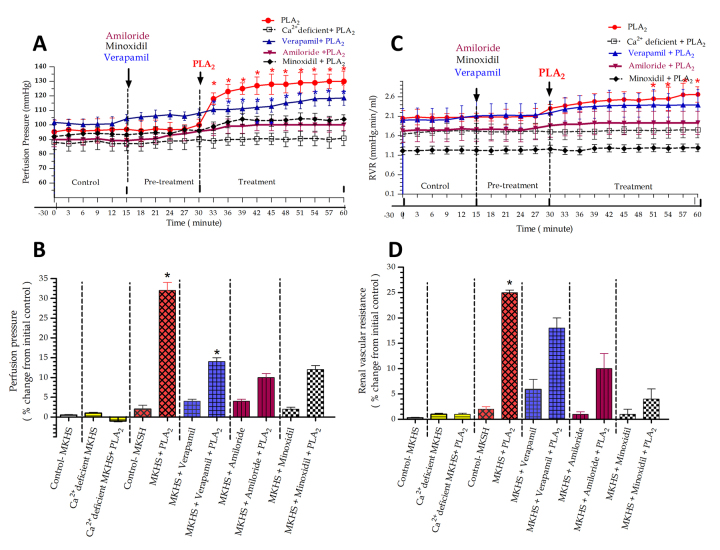
Time course of **(A)** PP and **(C)** RVR during 60 minutes of perfusion in five groups of four IPKs each. Each experiment was divided into control, pretreatment, and treatment periods. In each group, pretreatment with a specific ion channel blocker (verapamil, amiloride, or minoxidil) was followed by RvPLA₂ addition at 15 and 30 minutes, respectively. Each data point represents the mean ± SEM (n = 4). *p < 0.05 indicates a significant difference from the control period using ANOVA and Bonferroni’s post hoc test. In Figures 1B and 1D, maximal responses in percentage changes of **(B)** PP and **(D)** RVR are compared across groups. Average values were calculated over a 15-minute collection during the pretreatment period and a 30-minute collection during the treatment period, relative to control. Asterisks above the bars indicate significant differences from the control value at *p* < 0.05 in the same group using paired *t*-test.

**Figure 2:**
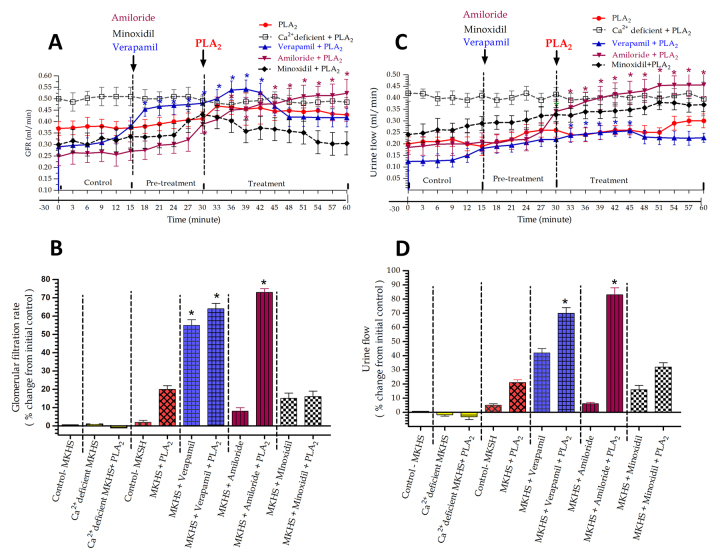
Time course of **(A)** GFR and **(C)** UF during 60 minutes of perfusion in five groups of four IPKs each. Each experiment was divided into control, pretreatment, and treatment periods. In each group, pretreatment with a specific ion channel blocker (verapamil, amiloride, or minoxidil) was followed by RvPLA₂ addition at 15 and 30 minutes, respectively. Each data point represents the mean ± SEM (n = 4). *p < 0.05 indicates a significant difference from the control period using ANOVA and Bonferroni’s post hoc test. In Figures 2B and 2D, maximal responses in percentage changes of **(B)** GFR and **(D)** UF are compared across groups. Average values were calculated over a 15-minute collection during the pretreatment period and a 30-minute collection during the treatment period, relative to control. Asterisks above the bars indicate significant differences from the control value at *p* < 0.05 in the same group using paired *t*-test.

**Figure 3:**
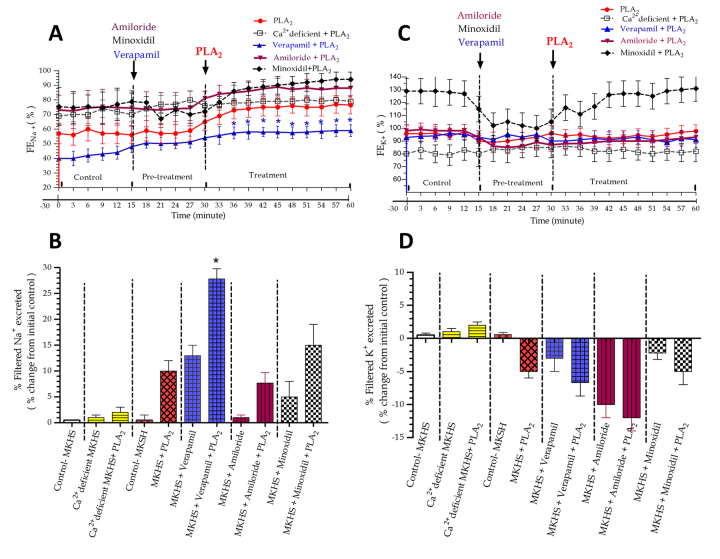
Time course of **(A)** FE_Na⁺_ and **(C)** FE_K⁺_ during 60 minutes of perfusion in five groups of four IPKs each. Each experiment was divided into control, pretreatment, and treatment periods. In each group, pretreatment with a specific ion channel blocker (verapamil, amiloride, or minoxidil) was followed by RvPLA₂ addition at 15 and 30 minutes, respectively. Each data point represents the mean ± SEM (n = 4). *p < 0.05 indicates a significant difference from the control period using ANOVA and Bonferroni’s post hoc test. In Figures 3B and 3D, maximal responses in percentage changes of **(B)** FE_Na⁺_ and **(D)** FE_K⁺_ are compared across groups. Average values were calculated over a 15-minute collection during the pretreatment period and a 30-minute collection during the treatment period, relative to control. Asterisks above the bars indicate significant differences from the control value at *p* < 0.05 in the same group using paired *t*-test.

**Figure 4:**
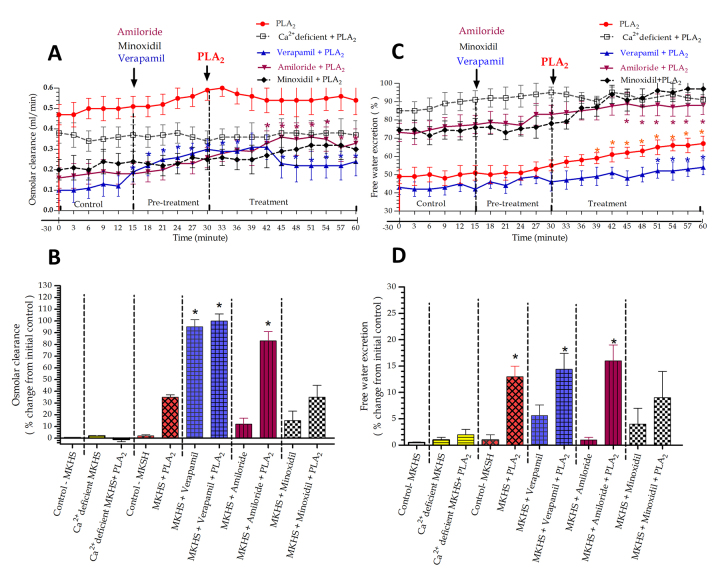
Time course of **(A)** C_osm_ and **(C)** free water excretion during 60 minutes of perfusion in five groups of four IPKs each. Each experiment was divided into control, pretreatment, and treatment periods. In each group, pretreatment with a specific ion channel blocker (verapamil, amiloride, or minoxidil) was followed by RvPLA₂ addition at 15 and 30 minutes, respectively. Each data point represents the mean ± SEM (n = 4). *p < 0.05 indicates a significant difference from the control period using ANOVA and Bonferroni’s post hoc test. In Figures 4B and 4D, maximal responses in percentage changes of **(B)** C_osm_ and **(D)** free water excretion are compared across groups. Average values were calculated over a 15-minute collection during the pretreatment period and a 30-minute collection during the treatment period, relative to control. Asterisks above the bars indicate significant differences from the control value at *p* < 0.05 in the same group using paired *t*-test.

Under the experimental protocol, the effects of RvPLA₂ on renal function in rabbit IPKs perfused with Ca²⁺-deficient MKHS were investigated. After an initial 30-minute equilibration period with Ca²⁺-deficient MKHS perfusion as the internal control, 1 mL of RvPLA₂ (280 μg/mL) was added to 100 mL of Ca²⁺-deficient MKHS perfusate. The changes in kidney functional parameters are presented as either the time course (Figures 1 to 4, panels A and C) or as average maximal responses in percentage from the initial control (Figures 1 to 4, panels B and D). Parameters such as renal hemodynamics (PP and RVR), glomerular filtration rate, urine flow, fractional excretion of Na⁺ and K⁺, and C_osm_ remained stable after the addition of RvPLA₂ throughout the perfusion period.

### Effects of RvPLA_2_ and pretreated with either verapamil, amiloride or minoxidil on the PP and RVR 

Results shown in Figure 1A reveal that in the group of IPKs treated with 1 mL of RvPLA₂ alone (280 μg/mL) in 100 mL of standard MKHS perfusate at 30 minutes of perfusion, there were initial increases in PP and RVR at 3 minutes after administration, followed by further significant stepwise increases (*p* < 0.05) throughout the perfusion time (Figures 1A and 1C). The maximal average percentage changes from initial control values for PP and RVR after administration of RvPLA₂ alone were 32% and 25%, respectively (*p* < 0.05) (Figures 1B and 1D).

In the group pretreated with verapamil alone (4 mg/100 mL), added at 15 minutes of perfusion, slight increases in PP (4%) and RVR (6%) from initial control values were observed (Figures 1B and 1D). After the addition of RvPLA₂ (280 μg/mL) at 30 minutes of perfusion, coexisting with verapamil pretreatment, PP and RVR increased at 3 minutes post-administration, followed by significant stepwise increases in PP (*p* < 0.05) and RVR compared to internal control throughout the perfusion period (Figures 1A and 1C). The combined effect of RvPLA₂ and verapamil resulted in increases of 14% for PP and 18% for RVR.

In the amiloride group (500 μg/100 mL), added at 15 minutes, slight increases in PP (4%) and RVR (1%) were observed from baseline. Following RvPLA₂ addition at 30 minutes, PP and RVR increased at 3 minutes, followed by further stepwise increases of 10% for both parameters compared to the internal control (Figures 1A to 1D).

In the minoxidil group (5 mg/100 mL), pretreatment at 15 minutes produced little change in PP and RVR. After RvPLA₂ addition at 30 minutes, slight increases in PP (12%) and RVR (4%) were observed (Figures 1A to 1D).

### Effects of RvPLA_2_ and pretreated with verapamil, amiloride and minoxidil on the GFR and UF in rabbit IPK

Administration of RvPLA₂ (280 μg/mL) alone into 100 mL of standard MKHS perfusate at 30 minutes of perfusion resulted in non-significant increases in both GFR and UF, approximately 20% above the initial control, which persisted until the end of the perfusion period (Figures 2A to 2D).

In the group of IPKs treated with verapamil (4 mg/mL) at 15 minutes of perfusion, there were significant increases in both GFR (55%, *p* < 0.05) and UF (approximately 42%) compared to the internal control. When RvPLA₂ (280 μg/mL) was added to the perfusate at 30 minutes, following pretreatment with verapamil, it resulted in marked and significant increases in GFR (64%, *p* < 0.05) and UF (70%, *p* < 0.05) throughout the remaining 60 minutes of perfusion.

In contrast, the group pretreated with amiloride alone (500 μg/mL) at 15 minutes of perfusion showed minimal effects on GFR and UF, with increases of approximately 6-8% compared to the initial control values. However, following the addition of RvPLA₂ (280 μg/mL) after amiloride pretreatment, there were significant increases in GFR (73%, *p* < 0.05) and UF (83%, *p* < 0.05) compared to the initial control values throughout the 60-minute perfusion.

In the group pretreated with minoxidil alone (5 mg/mL) at 15 minutes of perfusion, there were slight increases in GFR (15%) and UF (16%) compared to the initial control values. After this period, the addition of RvPLA₂ (280 μg/mL) in combination with minoxidil pretreatment resulted in non-significant increases in GFR (16%) and UF (32%) compared to the initial control values throughout the 60-minute perfusion.

### Effects of RvPLA_2_ and pretreated with verapamil, amiloride and minoxidil on the fractional excretion of Na^+^(FE_Na+_) and K^+^(FE_K+_) in rabbit IPK

The addition of RvPLA₂ alone (280 μg/mL) into 100 mL of perfusate at 30 minutes of perfusion resulted in a non-significant increase in FE_Na⁺_ of approximately 10%, while FE_K⁺_ decreased by about 5% compared to the internal control throughout the perfusion period (Figures 3A and 3C).

In the group of IPKs pretreated with verapamil alone (4 mg/mL) at 15 minutes of perfusion, there was a non-significant increase in FE_Na⁺_ (13%), while FE_K⁺_ decreased by approximately 3% compared to the internal control. When RvPLA₂ (280 μg/mL) was added at 30 minutes, following pretreatment with verapamil, it resulted in significant increases in FE_Na⁺_ (27%, *p* < 0.05), while FE_K⁺_ decreased by about 7% compared to the internal control throughout the remaining 60 minutes of perfusion (Figures 3B and 3D).

In the group pretreated with amiloride alone (500 μg/mL) at 15 minutes of perfusion, there was little effect on FE_Na⁺_ while FE_K⁺_ decreased by approximately 10% compared to the initial control values. After the addition of RvPLA₂ (280 μg/mL) following amiloride pretreatment, FE_Na⁺_ increased by 8%, while FE_K⁺_ decreased by approximately 12% compared to the initial control values throughout the 60-minute perfusion (Figures 3B and 3D).

In the group pretreated with minoxidil alone (5 mg/mL) at 15 minutes of perfusion, there was a slight increase in FE_Na⁺_ (5%), while FE_K⁺_ slightly decreased compared to the initial control values. After RvPLA₂ (280 μg/mL) was added, coexisting with minoxidil pretreatment, there were non-significant increases in FE_Na⁺_ (15%) and a slight decrease in FE_K⁺_ (5%) compared to the initial control values throughout the 60-minute perfusion (Figures 3B and 3D).

### Effects of RvPLA_2_ and pretreated with verapamil, amiloride and minoxidil on the C_osm_ and free water excretion in rabbit IPK

Administration of RvPLA₂ (280 μg/mL) alone into 100 mL of perfusate of standard MKHS at 30 minutes of perfusion resulted in non-significant increases in C_osm_ by approximately 35%, while free water excretion significantly increased (*p* < 0.05) compared to the initial control throughout the remainder of the perfusion (Figures 4A to 4D).

In the group of IPKs pretreated with verapamil (4 mg/mL) added to 100 mL of perfusate at 15 minutes of perfusion, significant increases in C_osm_ (95%, *p* < 0.05) were observed, while free water excretion increased by approximately 6% compared to the internal control. When RvPLA₂ (280 μg/mL) was added at 30 minutes, following pretreatment with verapamil, it resulted in a marked response with significant increases in both C_osm_ (100%, *p* < 0.05) and free water excretion (*p* < 0.05) throughout the 60-minute perfusion.

In the group pretreated with amiloride alone (500 μg/mL) at 15 minutes of perfusion, there was little effect on both C_osm_ and free water excretion compared to the initial control values. After this period, the addition of RvPLA₂ (280 μg/mL) to the perfusate, coexisting with amiloride pretreatment, resulted in significant increases in both C_osm_ (*p* < 0.05) and free water excretion (*p* < 0.05) compared to the initial control values throughout the 60-minute perfusion.

In the group pretreated with minoxidil alone (5 mg/mL) at 15 minutes of perfusion, slight increases in C_osm_ (15%) and free water excretion (4%) were observed compared to the initial control values. After the addition of RvPLA₂ (280 μg/mL) to the perfusate, coexisting with minoxidil pretreatment, non-significant increases in C_osm_ (35%) and free water excretion (10%) were observed compared to the initial control values throughout the 60-minute perfusion.

## Discussion

The phospholipase A₂ (PLA₂) component is a dominant protein family in the venom of all snake species [18] and a principal component of viperid venoms [19]. It has been extensively investigated for its pathophysiological effects [20], being rich in hydrolytic enzymes [21] and displaying considerable toxic heterogeneity, including neurotoxic, myotoxic, cytotoxic, anticoagulant, and inflammatory activities [22, 23]. The catalytic activity of PLA₂ induces acute local inflammation by hydrolyzing fatty acids at the sn-2 position of phospholipid membranes and subcellular organelles. Lysophosphatidylcholine, a byproduct of PLA₂ action, is a potent inflammatory mediator that modulates ROS production at inflammation sites [24].

Despite several studies on envenomation by crude *Daboia siamensis* venom in *in vivo* rabbit models [9] and in the isolated perfused kidney (IPK) system [6], the specific mechanisms by which PLA₂ induces acute kidney injury (AKI) remain unclear. These studies have reported nephrotoxic effects and alterations in renal parameters leading to AKI, but they do not directly evaluate the impact of PLA₂ - one of the main components of *D. siamensis* venom [9] - on renal ion channel function. Kidney function depends heavily on the regulation of electrolyte balance via ion channels localized in specific renal tissues. Our previous IPK studies showed RvPLA₂-induced histopathological changes, including crystal deposits in glomerular capillaries, dilation of tubular structures, and tubulonephrosis [7]. Ion channel dysfunction can severely disrupt renal electrolyte homeostasis, leading to renal disease [25]. Thus, this study aimed to elucidate how RvPLA₂ interacts with ion channels in renal cells and affects kidney function.

### The characterized effect of RvPLA_2_ on the renal function with Ca^2+^activation in rabbit IPK 

The IPK model offers a robust *ex vivo* platform to assess the direct effects of venom components on renal vascular and tubular functions, free from systemic influences such as hormones or circulating cells. The modified Krebs-Henseleit solution (MKHS) used here contained no ion-chelating agents [16], ensuring that systemic calcium-related feedback mechanisms would not interfere with the direct renal effects of RvPLA₂.

In IPKs perfused with Ca²⁺-deficient MKHS (lacking CaCl₂), the administration of 1 mL RvPLA₂ (280 µg/mL in PBS) into 100 mL perfusate resulted in no changes in PP, RVR, GFR, UF, or electrolyte excretion during the 60-minute period (Figures 1 to 4). These findings support that PLA₂ enzymatic activity is Ca²⁺-dependent [15]. This requirement is consistent with structural studies showing PLA₂s possess conserved calcium-binding loops (XCGXGG) and catalytic motifs (DXCCXXHD), where calcium coordinates with Asp49 and other residues at the active site [26, 27]. Additionally, basic PLA₂ subunits can disrupt membrane integrity through interactions with negatively charged phospholipids, increasing ion permeability [27, 28, 29].

Contrastingly, in IPKs perfused with standard MKHS containing 1.9 mM Ca²⁺, the same dose of RvPLA₂ (280 µg/mL) significantly increased PP and RVR and caused non-significant increases in GFR, UF, and osmolar clearance (C_osm_). Free water excretion significantly increased over the 60-minute period (Figures 1 to 4), likely due to the absence of ADH in this *ex vivo* model. These results align with our previous findings on venom fractions in IPK rabbits [7].

The observed hemodynamic changes may reflect RvPLA₂-induced alterations in membrane permeability, particularly in endothelial cells, potentially via platelet-activating factor (PAF) synthesis [30, 31]. PAF, a pro-inflammatory lipid mediator, influences renal hemodynamics and may cause vasoconstriction or vasodilation depending on concentration [32]. Increases in renal PAF have been associated with endothelial damage and vasoconstriction [32]. PAF antagonists such as WEB 2086 have reversed RvPLA₂-induced renal effects in prior IPK studies [7, 33, 34], and have blocked venom-induced GFR and UF reduction in rodent IPK models [35, 36]. However, PAF synthesis is absent in ATP-stimulated endothelial cells under Ca²⁺-free conditions [37, 38], further supporting the Ca²⁺-dependence of RvPLA₂ action.

Because the IPK lacks blood components, the increased PP and RVR observed here were not caused by coagulation. Instead, RvPLA₂ may induce endothelial cell lysis, triggering vasoconstrictor release, including PAF, though other mechanisms may also contribute. Further research is needed to explore PAF-independent effects.

The dose of RvPLA₂ used (280 µg/mL) corresponds to ~2.6-fold that present in the crude venom, considering PLA₂ comprises ~20-25% of *D. siamensis* venom [21, 39]. Such a high dose could intensify non-specific hydrolysis of phospholipids, affecting membrane-embedded ion channels. Ion channels are tightly regulated in specific nephron regions, and their dysfunction may disturb ionic gradients, alter osmotic balance, and contribute to AKI [40].

PLA₂-mediated membrane hydrolysis disrupts charge distributions and weakens van der Waals forces, damaging membrane structure [41]. This may lead to Na⁺ influx through increased permeability or via endothelial sodium channels (EnNaC), followed by depolarization and opening of voltage-gated Ca²⁺ channels, increasing intracellular Ca²⁺ concentrations [42-45]. Additional Ca²⁺ influx may result from Na⁺/Ca²⁺ exchange and release from sarcoplasmic reticulum stores [46]. These elevations in cytosolic Ca²⁺ activate smooth muscle contraction, increasing RVR and PP. Despite slight GFR and UF increases (Figures 2 and 3), these may be due to glomerular membrane damage, increasing permeability and inulin clearance.

RvPLA₂ may also impair energy metabolism. Previous studies have shown Na⁺/K⁺ ATPase activity is reduced in cortex and medulla following *D. siamensis* envenomation [47]. This reduction compromises tubular Na⁺ reabsorption, especially in segments utilizing epithelial sodium channels (ENaC) or Na⁺/H⁺ exchangers (NHE3), leading to osmotic diuresis and increased C_osm_ and free water excretion (Figure 4). Consequently, urinary Na⁺ excretion increased relative to GFR, elevating fractional sodium excretion (%FE_Na⁺_), while %FE_K⁺_ slightly decreased, consistent with findings in rat IPK models [48, 49]. These changes may relate to a decrease in Na^+^-K^+^ATPase activity [50].

### The effect of RvPLA₂ on renal function with pretreatment of Ca²⁺ channel blocker

Direct measurement of ion channel activity in biological membranes *ex vivo* is technically limited. Therefore, this study used ion channel blockers in the IPK model to assess whether RvPLA₂ affects renal ion transport indirectly, as such blockers are known to influence both vascular and tubular kidney functions [51-53]. Intracellular calcium accumulation has been implicated in acute renal failure pathophysiology [54], but how RvPLA₂ modulates Ca²⁺ transport remains unclear. Calcium channels are categorized into voltage-gated, voltage-independent, and intracellular release channels, such as ryanodine receptors, which are activated by Ca²⁺ or via allosteric coupling with plasmalemmal DHPRs [55, 56].

We investigated whether RvPLA₂ elevates intracellular Ca²⁺ by using verapamil, an L-type Ca²⁺ channel blocker [57-59]. Verapamil affects both vascular and tubular kidney functions [51-53]. We used a concentration of 4 mg/100 mL (8 × 10⁻⁵ M), which is sufficient to inhibit Ca²⁺ entry based on prior IPK [60] and non-excitable tissue studies [61, 62]. Voltage-gated Ca²⁺ channels use the electrochemical gradient to allow Ca²⁺ entry during membrane depolarization, mediated by voltage-sensing positively charged amino acids [56, 63].

Verapamil alone significantly increased PP, GFR, and C_osm_, with non-significant increases in RVR, UF, FE_Na⁺_, and FE_K⁺_ (Figures 1 to 4). It does not fully block Ca²⁺ entry, as alternative routes like Na⁺/Ca²⁺ exchange remain active [46]. These findings align with prior reports of verapamil enhancing GFR, UF, and electrolyte excretion [53]. In the kidney, L-type Ca²⁺ channels are on afferent arterioles, while T-type channels are on both afferent and efferent arterioles [64-66]. Verapamil dilates afferent arterioles, while T-type channel activity on efferent arterioles remains, increasing glomerular pressure and enhancing filtration.

In IPKs pretreated with verapamil, RvPLA₂ further elevated PP, RVR, GFR, UF, and electrolyte excretion (Figures 1 to 4). Although L-type channels were blocked, RvPLA₂ likely activated T-type channels [64, 67] or triggered intracellular Ca²⁺ release from stores [68-70], increasing cytosolic Ca²⁺ and inducing vasoconstriction via myosin light chain kinase modulation [71]. Additionally, RvPLA₂-induced endothelial lysis may release vasoconstrictors like PAF [7], overriding verapamil’s effects.

### The effect of RvPLA_2_ on renal function with pretreatment of Na^+^ channel blocker

Epithelial sodium channels (ENaC) regulate Na⁺ and water reabsorption in epithelial cells, while endothelial sodium channels (EnNaC) modulate endothelial stiffness and vascular tone [72, 73]. We evaluated whether inhibiting both ENaC and EnNaC activity with amiloride (500 µg/100 mL), a prototypical blocker, would mitigate RvPLA₂-induced dysfunction in the IPK model.

Amiloride alone did not alter RVR, PP, GFR, UF, or FE_Na⁺_ (Figures 1 to 4), suggesting minimal hemodynamic impact in this setting. This supports previous findings that amiloride softens the endothelial surface without major effects on renal blood flow [74].

Co-administration of RvPLA₂ and amiloride significantly increased PP, RVR, GFR, UF, FE_Na⁺_, C_osm_, and free water excretion, with decreased FE_K⁺_ (Figures 1 to 4). These findings suggest that RvPLA₂ exerts effects independently of ENaC and EnNaC inhibition, likely via membrane hydrolysis, elevating intracellular Na⁺ and Ca²⁺ and inducing vasoconstriction. Increased GFR and UF may result from efferent arteriole constriction or glomerular membrane damage, increasing permeability and inulin clearance (Figure 2).

RvPLA₂ and amiloride co-treatment may also impair Na⁺ reabsorption in distal nephron segments by blocking ENaC, producing natriuresis, increased FE_Na⁺_, C_osm_, and free water excretion (Figures 3 to 4). The associated Na⁺ retention in tubules may hyperpolarize membranes, reducing electrochemical gradients and suppressing K⁺ and H⁺ excretion, leading to decreased FE_K⁺_. Thus, RvPLA₂-induced vasoconstriction is distinct from ENaC and EnNaC inhibition, and amiloride appears insufficient to prevent the associated vascular damage.

ENaC channels regulate Na⁺ and water reabsorption and also modulate endothelial stiffness and vascular tone [72, 73]. We evaluated whether inhibiting ENaC activity with amiloride (500 µg/100 mL), a specific ENaC blocker, would mitigate RvPLA₂-induced dysfunction in the IPK model.

Amiloride alone did not alter RVR, PP, GFR, UF, or FE_Na⁺_ (Figures 1 to 4), suggesting minimal hemodynamic impact in this setting. This supports previous findings that amiloride softens the endothelial surface without major effects on renal blood flow [74].

Co-administration of RvPLA₂ and amiloride significantly increased PP, RVR, GFR, UF, FE_Na⁺_, C_osm_, and free water excretion, with decreased FE_K⁺_ (Figures 1 to 4). These findings suggest that RvPLA₂ exerts effects independently of ENaC inhibition, likely via membrane hydrolysis, elevating intracellular Na⁺ and Ca²⁺ and inducing vasoconstriction. Increased GFR and UF may result from efferent arteriole constriction or glomerular membrane damage, increasing permeability and inulin clearance (Figure 2).

RvPLA₂ and amiloride co-treatment may also impair Na⁺ reabsorption in distal nephron segments by blocking ENaC, producing natriuresis, increased FE_Na⁺_, C_osm_, and free water excretion (Figures 3 to 4). The associated Na⁺ retention in tubules may hyperpolarize membranes, reducing electrochemical gradients and suppressing K⁺ and H⁺ excretion, leading to decreased FE_K⁺_. Thus, RvPLA₂-induced vasoconstriction is distinct from ENaC inhibition, and amiloride appears insufficient to prevent the associated vascular damage.

### The effect of RvPLA_2_ on renal function with pretreatment of KATP channel opener

Potassium channels are essential for fluid balance and blood pressure regulation in the kidney [75]. K⁺ secretion occurs across nephron segments and involves voltage-gated, calcium-activated, and ATP-sensitive (K_ATP_) channels [76]. KATP channels are found in proximal tubule basolateral membranes, where they mediate K⁺ recycling via Na⁺/K⁺-ATPase [77].

This study evaluated whether KATP activation protects against RvPLA₂-induced dysfunction using minoxidil, a KATP opener and vasodilator [78, 79]. While clinically used for severe hypertension, minoxidil can cause Na⁺ retention and edema, possibly via increased Na⁺/2Cl⁻/K⁺ cotransporter activity in the thick ascending limb (TAL) [80].

Minoxidil alone caused no change in PP or RVR, with slight increases in GFR, UF, and FE_Na⁺_ and a small decrease in FE_K⁺_. Co-administration with RvPLA₂ led to increases in PP, RVR, FE_Na⁺_, C_osm_, and free water excretion, with further decreases in FEK⁺ compared to controls (Figures 1 to 4). These results indicate that RvPLA₂ exerts effects independently of KATP modulation, likely through ATP depletion and membrane damage that elevate cytosolic Na⁺ and Ca²⁺, disrupting cytoskeletal integrity and causing vasoconstriction and epithelial toxicity.

Overall, RvPLA₂ activity depends on Ca²⁺ and targets ion channels in glomerular and tubular membranes. Its effects contribute to AKI pathophysiology and are not ameliorated by ion channel blockers in the IPK model. Although clinical translation is limited due to the absence of systemic compensatory mechanisms, RvPLA₂-induced damage through hydrolytic action and membrane disruption is significant. RvPLA₂ also promotes redox imbalance and cytokine production [81].

Future studies should investigate early AKI interventions and assess the therapeutic potential of PLA₂ inhibitors to protect renal function during envenomation. Compounds like varespladib and its methyl ester, currently under evaluation for snakebite therapy [82], may offer promising approaches. A deeper understanding of RvPLA₂ enzymatic mechanisms is essential for developing selective and effective inhibitors.

## Conclusion

In this study, using the rabbit IPK model, we demonstrated that the RvPLA₂ fraction from *Daboia siamensis* venom contributes to the development of AKI, particularly via inflammatory mechanisms. Our findings indicate that RvPLA₂’s catalytic hydrolysis is driven by its interaction with extracellular Ca²⁺. This interaction alters membrane conductance and depolarization, especially affecting ion channel function in renal cell membranes. The initial enzymatic activity of RvPLA₂ disrupts phospholipid integrity, potentially damaging intracellular organelles and cellular membranes.

These observations may explain how moderate elevations in intracellular Ca²⁺ can promote tissue damage via PLA₂ activation, even though PLA₂ appears to have low calcium sensitivity at first glance. RvPLA₂ altered glomerular and tubular function in a manner not mitigated by pretreatment with ion channel blockers such as verapamil, amiloride, or minoxidil. This suggests that RvPLA₂ targets specific membrane sites, including ion channels, that are not adequately protected by standard therapeutic agents aimed at modulating inflammation or cell injury signaling pathways.

In snakebite envenomation, early-stage AKI is typically asymptomatic, making diagnosis and timely intervention difficult. Its pathogenesis involves multiple complex mechanisms, often requiring combination treatment strategies. While the *ex vivo* IPK model has limitations, it offers advantages in experimental control, organ consistency, and mechanistic insight.

Our results highlight the nephrotoxic potential of RvPLA₂ - the most abundant and toxic component of *D. siamensis* venom - particularly in the presence of extracellular Ca²⁺. Although some protective effects were observed with ion channel blockers in this experimental context, their efficacy in clinical scenarios remains uncertain. In real-life envenomations, larger venom doses and the presence of other toxic components may produce additional effects not captured here.

Therefore, combining ion channel receptor blockers with standard antivenom therapy may enhance the management of AKI following snakebite, particularly in preclinical settings. Future studies should focus on optimizing dosing regimens, using larger sample sizes, and applying more representative venom concentrations to better elucidate AKI mechanisms and evaluate potential therapeutic interventions.

## Abbreviations

ADH: antidiuretic hormone; AKI: acute kidney injury; C: clearance; C_in_: inulin clearance; C_osm_: osmolar clearance; DHPR: dihydropyridine receptor; ENaC: epithelial sodium channels; EnNaC: endothelial sodium channels; FE_K+_: fractional potassium excretion; FE_Na+_: fractional sodium excretion; GFR: glomerular filtration rate; In: Inulin; IPK: isolated perfused kidney; KATP channel opener: potassiumATP channel opener; MKHS: modified Krebs-Henseleit solution; PAF: platelet activating factor; PP: perfusion pressure; PLA_2_: phospholipase A_2_; RVR: renal vascular resistance; RvPLA_2_: Russell viper phospholipase A_2_; UF: urine flow rate. 

## Data Availability

All data generated and analyzed during this study are included in this published article
